# First case report of usefulness of 18F-FDG PET/CT in diagnosing typhlitis (an oncological emergency)

**DOI:** 10.1186/1470-7330-15-S1-P8

**Published:** 2015-10-02

**Authors:** ZA Khan, F AlSugair, R AlSalloum, AR AlNaim, M Abouzeid, A AlSugair

**Affiliations:** 1 Department of Radiology and Nuclear Medicine, King Faisal Specialist Hospital & Research Centre, Riyadh, Kingdom of Saudi Arabia; 2 Al-Imam University, Riyadh, Kingdom of Saudi Arabia

## Aim

Typhlitis (neutropenicenterocolitis) is a life-threatening condition with 50% mortality occurring in 3.5% of adult neutropenic patients. With no definitive physical findings, diagnosis is usually made with contrast-enhanced computerised tomography (CECT). We present the first-ever case of this diagnosis being made with 18F-FDG PET/CT.

## Methods

A 35-year-old lady was admitted with febrile neutropenia complaining of shortness of breath, productive cough and chest tightness. Having been diagnosed with acute myeloid leukaemia nine months ago, her disease had relapsed soon after allogeneic stem cell transplant from matched donor. A few diagnostic CECTs during her hospital stay showed findings in chest only confirmed to be due to invasive fungal and viral infections. Following some improvement, she was prescribed palliative subcutaneous cytarabine. The patient developed backache and neck pain with no findings on MRI. Due to continued fever and sepsis despite maximum treatment, PET/CT was requested by infectious disease team.

## Results

PET/CT showed moderate uptake (SUVmax 5.1) in the caecum and rectum which were swollen and oedematous on unenhanced CT. A diagnosis of typhlitis was made. There was hepatosplenomegaly and increased bone marrow activity. There were a few foci of abnormal mild uptake in distal right humerus and both distal femora, which possibly represented scattered early osteomyelitis. The patient unfortunately continued to deteriorate and died just under two weeks later.

## Conclusion

18 F-FDG PET/CT is a useful technique for diagnosis of typhlitis. Being hybrid, it provides both structural and functional information. Whole body coverage may show additional findings allowing comprehensive patient management.

## Consent to publish

Written informed consent for publication of their clinical details and/or clinical images was obtained from the patient/parent/guardian/relative of the patient.

